# 3D Printing of Pharmaceuticals and Drug Delivery Devices

**DOI:** 10.3390/pharmaceutics12030266

**Published:** 2020-03-15

**Authors:** Essyrose Mathew, Giulia Pitzanti, Eneko Larrañeta, Dimitrios A. Lamprou

**Affiliations:** 1 School of Pharmacy, Queen’s University Belfast, 97 Lisburn Road, Belfast BT9 7BL, UK; emathew01@qub.ac.uk (E.M.); giulia.pitzanti@unica.it (G.P.); e.larraneta@qub.ac.uk (E.L.); 2 Department of Life and Environmental Sciences (Unit of Drug Sciences), University of Cagliari, 09124 Cagliari, Italy

**Keywords:** 3D printing, bioprinting, additive manufacturing, computer-aided design (CAD), drug delivery, personalized medicine, pharmaceutics

## Abstract

The process of 3D printing (3DP) was patented in 1986; however, the research in the field of 3DP did not become popular until the last decade. There has been an increasing research into the areas of 3DP for medical applications for fabricating prosthetics, bioprinting and pharmaceutics. This novel method allows the manufacture of dosage forms on demand, with modifications in the geometry and size resulting in changes to the release and dosage behaviour of the product. 3DP will allow wider adoption of personalised medicine due to the diversity and simplicity to change the design and dosage of the products, allowing the devices to be designed specific to the individual with the ability to alternate the drugs added to the product. Personalisation also has the potential to decrease the common side effects associated with generic dosage forms. This Special Issue Editorial outlines the current innovative research surrounding the topic of 3DP, focusing on bioprinting and various types of 3DP on applications for drug delivery as well advantages and future directions in this field of research.

## 1. Introduction

Three-dimensional printing (3DP) has become one of the most innovative technologies in the pharmaceutical field. Within the last decade, there has been a significant expansion in the manufacture of drug delivery and medical devices. Additive manufacturing (AM) includes a variety of processes in which a layer-by-layer process is used to fabricate a solid object. The 3DP techniques, which can be used in the pharmaceutical field and are also covered under this editorial include Stereolithography (SLA), Selective Laser Sintering (SLS), inkjet-based 3DP, extrusion-based Fused Deposition Modeling (FDM) and Bioprinting. 3DP allows the potential for printing dosage forms on demand, with low cost and ease of use. AM is leading towards personalised medicine as the dosing and release characteristics of the drug delivery devices can be easily changed by altering the geometries of the 3D design using computer-aided design (CAD). The research outlined below and in this Special Issue addresses the current areas of research in bioprinting and 3D printing of drug delivery systems, focusing on the advantages and future directions in the use of AM in the pharmaceutical industry.

## 2. Fused Deposition Modelling (FDM)

FDM is one of the most commonly explored additive manufacturing methods due to the low cost of printers, print quality and ability to use drug-loaded filaments through hot-melt extrusion (HME). In this issue, the most common way of incorporating drug into the system was by creating drug-loaded filament created through HME with Chew et al. being the only exception, choosing to soak the polymer filaments in a drug-loaded solution. The articles within the Special Issue that used the FDM method of printing are outlined in this section.

Current implants are made with non-biodegradable polymers, which require surgical removal from the body after use. 3DP allows tailored dosage forms to be created specific to the patient and their condition, using biodegradable polymers, which can be broken down in the body into products that can be easily excreted. Dimensions of already marketed implants were used to create the hollow 3DP implants using filaments made from HME. Polylactic acid (PLA) and Poly(vinyl alcohol) (PVA) filaments were used to print hollow implants with a PVA window. Implants were loaded with a model compound by directly packing the powder inside. After in vitro testing, the implants showed the potential to adapt the dosage forms according to the material and implant designs used. Additional modification of the implant design with coatings was also explored in this paper by Stewart et al., showing the potential to extend the release profiles of the implants. The simplicity of the method outlined above allows the potential for easy set up within a clinical setting so such drug delivery devices can be printed on demand [1].

Giri et al. presented a novel approach to the fabrication of gastro-retentive floating tablets (GRFT) by using HME in order to create drug-loaded filaments that can be used on an FDM printer to create 3D printed tablets. GRFT has the ability to stay above the normal gastric content of the stomach for a prolonged period of time allowing them to overcome common issues with oral dosage forms such as unpredictable gastrointestinal transit and emptying times and metabolic degradation. Theophylline was used along with hydroxypropyl cellulose (HPC) matrix to create drug-loaded filaments using HME. Tablets with varying infill densities and shell thicknesses were created. The resulting tablets were assessed by determined the drug content, dissolution rates, physiochemical properties and floating behaviour. The developed FDM printed GRFTs possessed buoyancy for 10 h and had zero-order release kinetics. Overall, the 3DP tablets had better controlled release rates in comparison to current pharmaceutical manufactured tablets. This study highlights the potential for HME coupled with 3D printing to produce effective oral dosage forms of GRFTs providing greater versatility and a simplified manufacturing method in comparison to conventional methods [2].

Lamichhane et al. used FDM printing technology to develop floating gastro-retentive tablets with controlled release properties. The polymers used to create the pregabalin-loaded filaments were hypromellose acetate succinate (HPMCAS) and polyethylene glycol (PEG 400). FDM was used to create cylindrical tablets with varying infill densities. Differential scanning calorimetry (DSC), Fourier-transform infrared (FTIR) spectroscopy, X-ray powder diffraction (XRPD), and thermogravimetric analysis (TGA) were used to evaluate the properties of the resulting tablets, showing that extrusion and printing process does not affect drug stability and crystallinity. It was shown that the presence of a top/bottom layer determined the floating capabilities of the printed tablets. In particular, closed systems retain the buoyancy for >24 h, open systems, on the contrary, failed to float. Moreover, open systems with a low infill percentage showed a faster drug release compared to open and closed systems with a high infill ratio. Thus, they optimised the formulation designing a tablet with a closed bottom layer and a partially opened top layer. This formulation showed a zero-order drug release and retained its floating ability for 24 h. This study showed that FDM printing technology is a suitable technology for the development of floating gastro-retentive tablets [3].

HME was used to also create HPC and ethyl cellulose-based filaments, which were used as a feedstock for printing the shells using FDM. Directly compressed theophylline tablets were used as the core. Theophylline is used forthe treatment of symptomatic treatment of chronic asthma. The aim was to develop a tablet with floating capabilities as well as the ability to have pulsatile release after the relative lag time to be able to improve the therapy for patients with asthma. The FDM printed shells varied in shell thickness, infill density and wall thickness. All of the geometries of printed core-shell tablets showed good floating capabilities with no lag time as well as good dissolution properties. This meant that lag time could be varied from 30 min to 6 h according to the requirement. The proportion of EC in the formulation had a significant effect on the floating lag time. The proposed floating pulsatile system showed promise for effective delivery of drugs that require a high residence time in the stomach. This method of drug delivery minimises adverse effects and can increase patient compliance. This method of printing also allows the use of thermolabile drugs to be delivered through this system, as the inner core is not directly exposed to the high temperatures of FDM printing [4].

Chew et al. used 5-aminosalicyclic acid (5-ASA) and fluorescein sodium (FS) to create fixed dose combination solid dispersion drug products. The drugs were impregnated into PVA filament by soaking the filament in a drug solvent mixture for 24 h, the filament was then heated to achieve rapid solvent evaporation. The influence of the use of different solvents during drug loading was also studied in order to understand the optimal drug loading as well as the mechanical and physiochemical properties of the filaments prior to printing the drug using FDM 3D printing. The results showed that the drug loading could be improved up to 3 times according to the choice of solvent due to its polarity. In this paper, it was proven that methanol (MeOH) had better properties as the solvent in comparison to ethanol (EtOH) for FS and 5-ASA. The resultant 3DP product using both drugs were solid dispersion fixed dose combination with favourable release profiles and behaviours [5].

Arany et al., produced cylindrical plates to test chemical, physical, mechanical and biological properties of 3DP materials. The plates were developed through FDM 3DP technology. First, PLA plates were printed and then the PLA plates were chemically modified with the addition of positive charged groups in order to decrease their lipophilicity. The material was characterised to assess the effective surface modification and the increased hydrophilicity. The MTT assay performed in Caco-2 cells demonstrated the cytocompatibility of the material. Moreover, the 3D printed plates showed an antifungal activity against Candida albicans. This work put the basis for further studies in the chemical functionalization of 3D printed materials [6].

Domínguez-Robles et al., used HME to develop antioxidant PLA filaments containing Lignin (LIG). In addition, tetracycline (TC), which has known antibiotic and antioxidant capabilities, was combined with PLA and LIG. The addition of LIG leaded to a filament with lower resistance to fracture. Moreover, as the LIG content was increased the materials showed a reduction in their wettability properties. The 2,2-diphenyl-1-picrylhydrazyl (DPPH) assay showed the antioxidant properties of the PLA filament containing LIG. The antimicrobial properties of the material were examined through bacterial adhesion studies performed on *S. aureus*. The addition of only LIG did not confer any antimicrobial properties to the material but the further addition of TC significant redacted the bacterial adherence. PLA-LIG filament was used to develop meshes for wound healing applications. Curcumin (CUR) was selected as a model drug and applied on the top of the mesh. The permeation studies showed that the grid size of the mesh affects the release rate of CUR. Small size leaded to a slower release. The combination of the mesh with a soluble PVA film further delayed the release rate. Accordingly to this study, PLA-LIG filament can be used to print personalised meshes for wound dressing applications [7].

Pelvic Organ Prolapse (POP) and stress urinary incontinence (SI) are currently treated with surgical implantation of vaginal meshes, with these conditions affecting 30–40% of women worldwide. These mesh reinforcements are often made from poly(propylene) (PP) or polyester, which have been approved by the Food and Drug Administration (FDA) for vaginal applications. However, since their approval there has been an increased number of complications associated with vaginal meshes, some of which include chronic pain, infection and high inflammatory response. Domínguez-Robles et al., proposed the use of an alternative material for these meshes, the Thermoplastic polyurethane (TPU), which is more flexible in nature. FDM was used to print meshes using Levofloxacin (LFX) loaded TPU filaments created using HME. The printed meshes were characterised using fracture force studies, FTIR, SEM, X-ray microcomputed tomography (µCT), release studies and bacterial testing. The µCT showed the LFX was evenly distributed throughout the TPU matrix at all the concentrations of drug used. Mechanical testing also revealed that the currently used PP is more rigid in nature compared to TPU and therefore, TPU is more suitable for vaginal meshes due to its elasticity. The printed TPU meshes also showed significant bacteriostatic activity against both *Staphylococcus aureus* and *Escherichia coli* cultures meaning infection rates associated with vaginal meshes could be reduced. This study effectively showed the use of 3DP as a novel approach for producing anti-infective drug loaded vaginal meshes with better mechanical properties in comparison to the PP meshes that currently being used [8].

Tidau et al., developed high-loaded theophylline filaments using a co-rotating twin-screw extruder. The polymers selected were polyethylene oxide and hypromellose (HPMC). Afterwards, the filaments were utilised to develop tablets of three different geometries (cylinders, rings and spheres) with fused layer modeling (FLM). The size and the morphology of the particles of the raw material were investigated. The filaments and the dosage forms were characterised in terms of mechanical properties, surface morphology, mass and content uniformity. A dissolution test was performed in order to evaluate the release rate of theophylline from the different dosage forms. The results showed that for HPMC in particular the larger load of theophylline resulted in increased flexural strength; however, the accuracy of the printing process decreased resulting in defects in the microstructure. No significant effect on the dissolution profiles was observed by increasing the load of theophylline. The work highlights how the amount of API used influence the 3DP process [9].

Alhijjaj et al., highlighted the need for a greater understanding of the process parameters of AM required for the development of 3DP from a group of proof of concept studies to an advanced tool used in the manufacture of pharmaceuticals. In this study, the processing parameters of FDM printing were explored. The macroscopic and microstructural reproducibility of the FDM process was examined, showing that the prints poorly matched the target dimensions, an issue that was also highlighted by Healy et al., in the SLA printing process. Weights of the printed grids was also another area that was highlighted in this study, with the weights varying according to print speed. The temperature of the print however, showed to have little effect on the print weight. A principal component analysis showed that there is an interaction between the accuracy of the weight and/or dosing and the dimensional authenticity, indicating the need for a compromise between these quality parameters. Summed Standard Deviation (SSD) was used as measure of the goodness of printing conditions, which can produce the best overall printing reproducibility, which was calculated by adding together all the standard deviations of each measured value for every condition with the lowest SSD value showing the most reproducible prints. In this study, the processing parameters of 120 °C and 90 mm/s had the lowest SSD, indicating that these were the parameters with the most reproducible prints [10].

## 3. Three-Dimensional Bioprinting

Three-dimensional bioprinting, includes all the variety of 3DP modes and is been used in different pharmaceutical studies and tissue engineering. Current treatments for bone fractures and bone defects involve bone grafts or metal prosthetic implants, which can be restrictive because of the substantial loss of tissue from surgery, prolonged recovery periods and donor site morbidity. Therefore, there is a need for a novel method of treatment for bone fractures and defects. Kondiah et al., explored 3D bio-printed pseudo-bone drug delivery scaffolds that have been fabricated from polypropylene fumarate (PPF), free radical polymerised polyethylene glycol-polycaprolactone (PEG-PCL-PEG) and Pluronic (PF127). The scaffolds were optimised using MATLAB software and artificial neural networks (ANN) with the ANN optimised scaffolds showing controlled release of Simvastatin for over 20 days. Simvastatin was incorporated into the scaffold due to its abilities to promote bone healing and repair. The bio-printed scaffolds were tested on fracture-induced human clavicle bones. The matrix analysis showed that after the application of the scaffolds, the fractured bone had similar matrix hardness and matrix resilience to healthy human clavicle bones. This highlights the potential for bioprinted pseudo bone scaffolds to fill in fracture sites, resulting in great adhesion of the fractured bone and restoration to intended mechanical strength [11].

Andriotis et al. developed a 3D bioprinted wound dressing using pectin-based bio ink. The properties of the pectin bioinks were optimised by the addition of chitosan and cyclodextrin inclusion complexes with propolis extract to improve the antimicrobial and wound healing properties of the inks. The inks were able to form transparent films when dried and exhibited fast disintegration upon contact with aqueous media. The in vitro wound healing studies showed that the addition of cyclodextrin/propolis extract inclusion complexes (CCP) enhanced wound healing as well as the antimicrobial properties of the patches, with a 95% increase in antimicrobial activity of the films. Addition of CCP up to a certain point also enhanced the bio-adhesive properties of the dressing. However, in higher concentrations of CCP, the cell viability was reduced >10% w/w. This may have been due to the presence of insoluble film material in the higher concentrations of CPP, which could physically obstruct the cells. Overall, this study was able to effectively show the potential for use of biodegradable, 3D printable inks for fabrication of direct and indirect wound dressings [12].

## 4. Stereolithography (SLA)

Healy et al., used SLA as the AM process to create oral dosage forms of 2.5% and 5% concentration of aspirin and paracetamol. A novel photopolymerisable resin was used and drugs were printed using an SLA 3D printer. Healy et al., were able to fabricate 28 drug dosage forms in one print cycle, showing the potential for bulk manufacture of oral dosage forms through SLA. This study also highlighted the effect of addition of drug on the dimensions of the printed dosage forms, with the printed form dimensions being different to the design. This highlights an area, which requires research, in the future, on how addition of materials can affect the printed product. The results from release studies showed that there was an increased in release of active drug when drug loading was increased, this highlights the potential for patient specific drugs to be created with the ability to modulate drug release. Overall, this study effectively highlighted the potential for creating solid dosage forms using SLA printing, with the research leading towards the ability to create personalised medication and the ability to modulate drug release from printed products [13].

Robles-Martinez et al., were able to construct a novel SLA printing method that allowed the production of multi-layered tablets (polypills) that had flexible drug content and shape. The drugs chosen for the work were paracetamol, caffeine, naproxen, chloramphenicol, prednisolone and aspirin. Three different tablets shapes were printed: cylinder, ring, and ring with a soluble filler. Raman microscopy confirmed the spatial separation of the drugs but also showed the ability of certain drugs (naproxen, aspirin, and paracetamol) to diffuse between the layers due to its solid-state characteristics. Dissolution tests showed that the polypill geometry and the type of excipient affected the drug release allowing distinct release profiles for each of the six drugs. This study showed the possibility to use SLA 3DP for fabricating multi-drugs tablets to improve personalisation for patients [14].

## 5. Other forms of Additive Manufacture

### 5.1. Selective Laser Sintering (SLS)

Similarly to SLA, this method of AM works with lasers but powder materials are fused together, whereas SLA works with a resin. Awad et al., utilised SLS 3DP, for the first time, to produce small oral dosage forms with modified release properties. They fabricated single miniprintlets using paracetamol as a model drug and dual miniprintlets where paracetamol is combined with ibuprofen. For the single miniprintlets, ethyl cellulose (EC) was employed as the main polymer matrix. In the case of dual miniprintlets one layer contained EC for sustained release whereas the second layer containing Kollicoat IR (a graft copolymer comprised of PEG: PVA, 1:3) for immediate release. In order to assess the effect size has on dissolution properties, miniprintlets of two different diameters, 1 mm and 2 mm, were developed. The single miniprintlets exhibited slow paracetamol release, which was reduced when increasing the diameter. For the dual miniprintlets, the diameter does not affect the paracetamol release profile. This work demonstrates the possibility to use SLS 3D printing to combine multiple Active Pharmaceutical Ingredients (APIs) with distinct release properties in a single dosage form [15].

### 5.2. Digital Light Processing (DLP)

DLP is another method of 3DP, which is similar to SLA. Is a resin-based method, however, rather than using a laser-focused UV beam, DLP uses UV light from a projector to cure each layer of the 3D printed product. Madzarevic et al., prepared ibuprofen tablets through DLP 3DP technology. Eleven formulations were prepared following the D-optimal mixture design from Design Expert software. It was noticed that an increase of water content leads to an enhancement of printing time. Two artificial neural networks (STATISTICA 7.0 and MATLAB R2014b) were used in order to evaluate how the components and the printing parameters affect the ibuprofen release. The data obtained from these two software were compared with the one obtained experimentally. The drug release predicted with STATISTICA 7.0 was quite similar to the one obtained experimentally. This study described that suitable ANN allows to recognise the input–output relationship in DLP printing of pharmaceutics [16].

### 5.3. Stencil Printing

Wickstrom et al., propose a new method of printing that has not been used in the production of pharmaceuticals. This study aimed to evaluate the feasibility of creating drug containing polymer inks, which could be used in the manufacture of flexible dosage forms, with products having acceptable content of uniformity and mass. Haloperidol (HAL) discs were printed using a prototype stencil printer, with polyester used as the stencil material. The stencil geometry was used to define the dose, with doses being altered by changing aperture areas and stencil heights. The therapeutic HAL was successfully created for treatment of 6–17 year-old children that fulfilled both mass and content of uniformity requirements. HAL dose was achieved by using 16% hydroxypropyl methylcellulose (HPMC) and 1% of lactic acid. The results show that the drug was amorphous after printing and the pH remained above pH 4. Disintegration studies showed that the orodispersible discs printed showed disintegrations times below 30 s. Therefore, it was concluded that the novel method of batchwise stencil printing, could be used as a viable method for the production of pharmaceuticals [17].

### 5.4. Embedded 3D Printing (e-3DP)

Embedded 3D printing is a novel form of AM in which a viscoelastic ink is extruded into a solidifying reservoir using a deposition nozzle at a predefined path. Rycerz et al., presented one of the first examples in using e-3DP in the pharmaceutical field to fabricate chewable oral dosage forms with dual drug loading. The two drugs used were paracetamol and ibuprofen, which were suspended in locust gum solution and an embedding medium of a gelatin-based matrix material. These were printed at an elevated temperature of 70 °C and then solidify at room temperature. The dosing of the printed dosage forms were varied by specifically altering the printing patterns. The rheology, printing speed and the needle size of the embedded phase were examined. This proof of concept study showed the potential for e-3DP to be used to print oral dosage forms that could include various materials, allow personalised dosing and geometry for novel oral dosage forms in paediatrics [18].

### 5.5. Semisolid Extrusion Printing (EXT) and Inkjet Printing (IJP)

Öblom et al., compared the conventional manufacturing method to produce patient-tailored doses of the anticoagulant drug warfarin at Helsinki University Hospital (HUS) Pharmacy, Finland’s largest hospital pharmacy, with two innovative printing techniques, named semisolid extrusion 3D printing (EXT) and inkjet printing (IJP). The printed orodispersible films (ODFs) showed a good thickness and flexibility and a better uniformity than the oral powders in unit dose sachets (OPSs). OPSs and ODFs remained stable for a period of one month and were appropriated for being given to the patients through a naso-gastric tube. In order to provide additional information about the dosage form and to reduce the errors that occur in the administration of medication a Quick Response (QR) code was printed onto the ODFs using IJP. The study demonstrated how printing technologies are promising techniques for the development of patient-specific dosage forms [19].

## 6. Critical Literature Reviews

This Special Issue also includes three review articles on the areas of FDM and 3DP as a whole. Azad et al., highlighted that is important to find out which polymers are appropriate for pharmaceuticals extrusion-based 3DP, focusing on FDM and pressure-assisted microsyringe (PAM). They also evaluated how the printing operations are affected by the material properties and by the comportment of the mixture of polymer and active component. They confronted 3DP with the current process of direct compression and evidenced the importance to know the rheological properties of the polymers and polymer–API mixture in order to predict their impact on the printing process and on the final dosage form. They also discussed which kind of characterisation methods are required for 3D printed structure, drug, polymer and other functional excipients. Finally, examined the challenges and opportunities related to pharmaceutical extrusion-based 3Dp [20].

The review of Kjar and Huang first described the different AM techniques and the resolution related to each technique described. They discussed the application of 3DP technology in the pharmaceutical field specific to micron sized manufacture. Finally, they highlighted the challenges and opportunities related to the use of AM in drug delivery and development with the main challenges outlined being gaining approval from regulatory authorities as well as changing from small- to large scale-manufacture of 3D printed products [21].

Araújo et al., aimed to present the possibility of turning current pharmacies into digital pharmacies with the ability to print dosage forms for patients on demand. The versatility of using FDM 3DP was explored, referring to HME as mentioned in previous papers using FDM, as an effective method for the addition of drugs allowing customizable dosage forms. Pharmaceutical companies will need to work in unison with these digital pharmacies for the large-scale production of extruded filaments, which the digital pharmacies can later use to print. The regulatory concerns surrounding 3DP were also evaluated showing that patents have hindered 3DP products from reaching the market and there should be greater cooperation between regulatory and patenting authorities to ensure that 3DP products can reach the market [22].

## 7. Conclusions/Future Directions

There is an increasing research using multiple types of 3DP. However, one of the main advantages common to all available types of 3DP is the ability to create personalised medicine. AM, due to its ease of use, speed and accessibility is increasingly promoting the creation of medical devices and pharmaceutical products on demand for patients within a clinical setting. The ability to change the release profile and dosing of a 3D printed tablet simply by changing the geometries using CAD and the ability to incorporate drugs into FDM printed injectable devices or mesh implants using HME, opens up a wide range of possibilities within the application of 3DP in the medical field.

However, more research does need to be conducted in the field before the production of 3D-printed products on demand can become a reality within a clinical setting, such as the effect of process parameters on the print quality and how reproducibility in 3DP can be improved. FDM is also limited to the number of drugs that can be loaded into filaments, as they need to withstand the high temperatures of the process. However, if research continues to rise in the area of 3DP, due to the versatility of 3D printed products and the number of manufacturing advantages that 3DP offers there is potential for more 3DP to leave the proof of concept stage and be developed into a widely used manufacturing tool.

The number of manuscripts published in this Special Issue focused on 3DP of oral dosage suggests an increasing interest in personalised medicine. Additionally, this Special Issue included several works describing the use of 3DP for other applications such as medical devices. Therefore, 3DP can be applied in a wide variety of fields within biomedical sciences. However, before this technology can be extensively applied for medical applications, multiple regulatory questions should be addressed. One of the main unanswered questions is the quality assurance if the dosage form/medical device is created on demand for each patient. In order to accelerate the acceptance of this technology, the US FDA published guideline documents for medical devices manufacturing using 3DP technology. Accordingly, we anticipate that more 3D printed pharmaceutical/medical products will reach to the market within the next few years.
